# Pilot Study Investigating Effects of Changing Process Variables on Elastic and Energy-Absorbing Characteristics in Polyurethane/Agglomerated Cork Mix for Use in Micro-Transport Helmet

**DOI:** 10.3390/ma17081925

**Published:** 2024-04-22

**Authors:** David E. White, Hyun Chan Kim, Mohammad Al-Rawi, Xiaowen Yuan, Tony Sojan

**Affiliations:** 1 School of Engineering, Computer and Mathematical Sciences, Auckland University of Technology, Auckland 1142, New Zealand; tonythengumpally@gmail.com; 2 Centre for Engineering and Industrial Design, Waikato Institute of Technology, Hamilton 3240, New Zealand; chan.kim@wintec.ac.nz (H.C.K.); mohammad.al-rawi@auckland.ac.nz (M.A.-R.); 3 Faculty of Engineering, Chemical and Materials Engineering, The University of Auckland, Auckland 1010, New Zealand; 4 Future Fibres Laboratory, School of Engineering, Computer and Mathematical Sciences, Auckland University of Technology, Auckland 1142, New Zealand

**Keywords:** polyurethane, agglomerated cork, mechanical properties, micro-transport

## Abstract

This pilot investigation identifies the influence that changing the process variables of curing pressure, curing temperature, and mix ratio of a polyurethane/agglomerated cork matrix has on the mechanical properties of energy absorption, Young’s modulus of elasticity, and spring stiffness in safety helmets intended for micro-transport riders. The results are compared to expanded polystyrene, a material commonly used in micro-transport helmets. Mechanical testing of the various samples found that, over the range tested, curing pressure had no effect on any of the mechanical properties, while increasing amounts of resin caused a stiffer structure, and increasing curing temperature led to increased energy absorption. Consistent with the elastic modulus findings, all polyurethane/agglomerated cork test samples demonstrated higher median levels of spring stiffness, ranging from 7.1% to 61.9% greater than those found for expanded polystyrene. The sample mixed at a 1.5:1 binder/cork ratio and cured at 40 °C displayed the closest spring stiffness to EPS. While the mechanical properties of the eco-friendly polyurethane/agglomerated cork matrix did not match those of expanded polystyrene, the difference in performance found in this study is promising. Further investigation into process variables could characterise this more ecologically based matrix with equivalent energy-absorbing and structural characteristics, making it equivalent to currently used expanded polystyrene and suitable for use in micro-transport helmets.

## 1. Introduction

E-scooters are widely used as new method of micro-transport due to their low cost, ease of access, availability, and versatility in crowded cities. Despite this popularity, over a four-month period in 2019, during the early stages of micro-transport options being introduced in the city of Auckland, New Zealand, 180 people presented to Auckland City Hospital Emergency Department as a result of e-scooter accidents, with one-third being admitted or transferred [1]. From this cohort, 65.6% had contusions and lacerations, 41.7% had fractures, and 17.2% received head injuries. Surprisingly, only three of these patients (1.6%) wore a helmet. During this time period, and despite the obvious head injury risk, the local government authority, Auckland City Council, implemented the recommendation that it ‘should not seek to implement a mandatory helmet law in isolation of other safety efforts’ [2]. Despite this policy, many micro-transport companies in New Zealand offer a shared helmet with e-scooter hire, and in many other cities worldwide, riders are often required to wear protective headwear to protect users from the risk of serious head injury [3,4,5,6]. Also, the helmet size is not easily portable for e-scooter users, and polymer-based materials are not sustainable. Therefore, designing a resizable and sustainable helmet based on a circular economy approach is needed.

The challenge facing micro-transport hire providers is the reluctance of users to wear the shared helmet available on the rental e-scooter for a variety of reasons, including poor hygiene or fit [7]. With a growing environmental awareness, micro-transport users are now looking to own their personal mobility helmet made from eco-friendly materials compared to the current types using synthetic materials that present significant environmental issues when disposed [8].

The New Zealand micro-transport helmet safety standard, AS/NZS 2063:2020, sets design objectives that include ‘diminishing the effects of a blow to the head from a person riding or falling from a bicycle or wheeled recreational device’ [9]. This is realised through both structural design and the careful selection of the helmet material to absorb impact energy and distribute force. These attributes are assessed by impact energy, load distribution, and dynamic strength tests prescribed by this standard [9]. The most commonly used energy-absorbing material used in micro-transport helmets is expanded polystyrene (EPS), a light-weight petroleum product that is commonly used in structural applications due to its favourable energy-absorbing [10] and mechanical structural characteristics [11]. Unfortunately, EPS does not currently have a clear post-use recycling pathway, and therefore, its disposal has negative environmental impacts, where the USA alone produces 7190 tons of EPS waste per annum which releases greenhouse gases when composted or burnt [11]. A recent computational study of micro-transport helmets has identified more sustainable materials, including paper and cork, as having mechanical properties superior to EPS [8]. Cork is a naturally produced material, sourced from the bark of the Oak tree (*Quercus suber* L.), that has many desirable mechanical properties for use in a micro-transport helmet, including the capacity to absorb energy with high strain levels without mechanical failure [12]. Large amounts of cork are produced as waste from stopper production that can be granulated and mixed with a binder to form agglomerated cork for use in a variety of value-added applications where energy absorption is desired, such as shoe soles or floor tiles [12]. Previous testing has demonstrated that aromatic polyurethane (APU) and micro-agglomerated cork (<3 mm particle size) are potentially an ideal combination for replacing EPS in micro-transport helmets given the combination of a smaller particle size and aromatic binders forms a composite structure more than twice as elastic and energy-absorbing as structures formed from coarser grain sizes and aliphatic binders [13]. While synthetic polyurethane materials are sourced from petrochemical-based raw materials and are therefore not biodegradable, these have now been replaced by bio-based polyurethanes (PUs) that are biodegradable [14]. The use of PU binding resin provides additional benefits of water resistance compared to other common binders such as melamine–urea–formaldehyde [15].

Variation in the process variables of the binder-to-cork ratio, curing pressure, and curing temperature could potentially influence the energy absorption and other mechanical properties of the final matrix [12,16]. While recent research into helmet impact energy absorption has shown that a combined agglomerated and expanded (black) cork matrix demonstrates a 10% reduction in peak force transfer compared to agglomerated cork [17], the authors were unable to find any other studies investigating the change in mechanical properties caused by variations in the agglomerated cork matrix processing parameters. This investigation seeks to identify the process variables of micro-agglomerated cork bound with aliphatic polyurethane to inform the production of an environmentally friendly alternative to the use of EPS in micro-transport helmets. This will be achieved by controlling the process variables of the PU binder-to-cork ratio, curing pressure, and curing temperature to produce an environmentally friendly composite structure that will be compared to an EPS sample taken from a commercially available micro-transport helmet. This will be performed experimentally using an energy absorption test rig for specimens produced using different curing temperatures and binder/cork mix mass ratios to determine the Young’s modulus of elasticity and spring stiffness.

## 2. Materials and Methods

For the purposes of this pilot study, supply issues constrained us to using biodegradable cork and an available synthetic two-part, aliphatic-based polyurethane elastomer that did not contain Methylene bis 2,4 aniline (MOCA), Toluene diisocyanate (TDI), or 4,4′-Methylenedianiline (MDA).

### 2.1. Sample Preparation

**Figure 1:**
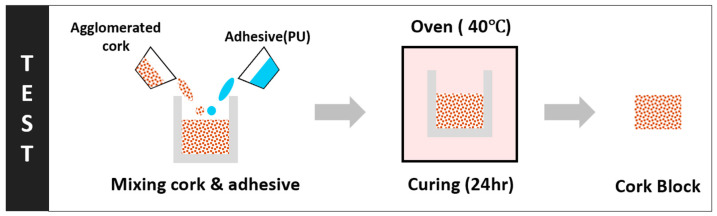
The binder for cork: polyurethane resin to control the binder/cork mix with gray arrows indicating stages of processing.

Mix mass ratios of both cork (2–3 mm, Amorim Cork, Santa Maria de Lamas, Aveiro, Portugal) and the two-part colourless polyurethane elastomer (WC565, Barnes, New Zealand) were carefully measured using precision scales prior to mixing at the predetermined binder/cork mix mass ratios given in Table 1, as shown in the schematic diagram of the preparation process (Figure 1).

**Figure 2:**
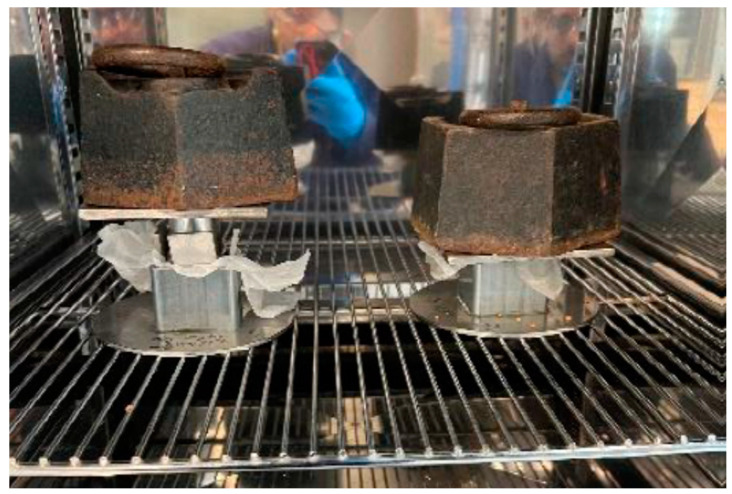
Sample mould containing compressed binder/cork mixture within curing oven.

Each of the different binder/cork mix mass ratios were poured into an open rectangular steel mould, 43 mm square and 24 mm high, before a weighted mass was placed on top of the setting mixture to push out air from the mixture. The whole assembly was then placed into a preheated oven to cure, as shown in Figure 2.

Combinations of different binder/cork masses and their respective mass mixture ratios, setting forces and pressures, and setting temperatures used in this investigation are also given in Table 1.

**Figure 3:**
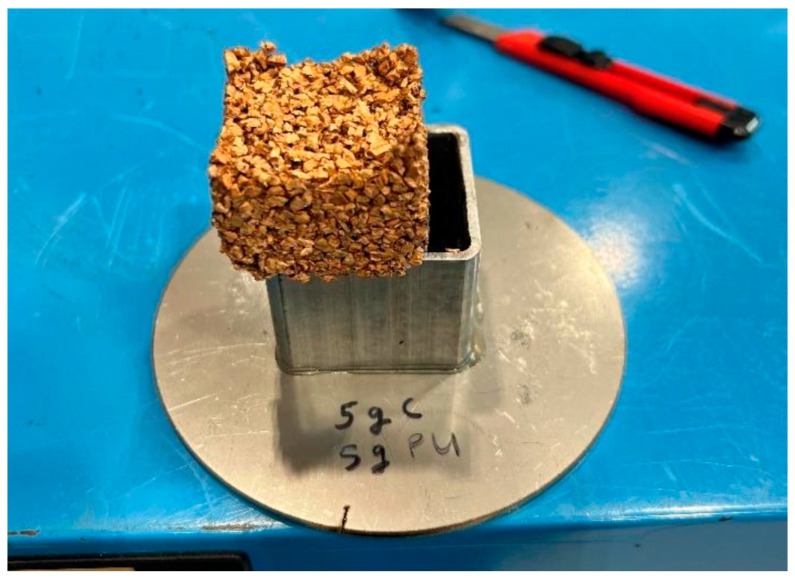
Polyurethane/cork test specimen, mixed at 1:1 ratio of 5 g cork and 5 g polyurethane, removed from mould.

After setting for 24 h under controlled pressure in a temperature-controlled oven, the test specimens were removed from the mould, as shown in Figure 3, and allowed to cool to room temperature before the upper and lower test surfaces were ground flat to a height of 24 mm using a material testing surface grinder in preparation for mechanical testing. The main reason for the long curing duration was to ensure that the specimen was dry before the experiment and to avoid stickiness.

### 2.2. Benchmarking Material Properties

**Figure 4:**
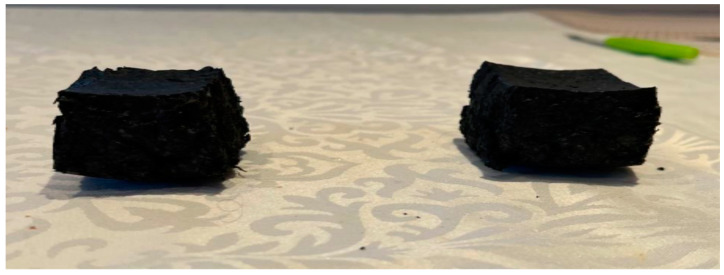
Expanded polystyrene test specimens extracted from scooter helmet.

For benchmarking purposes, expanded polystyrene test specimens of identical size to those produced though compression moulding of PU and cork, shown in Figure 4, were extracted from a commercially available e-scooter helmet. These benchmarking specimens were then prepared for testing in the same manner as previously described for the PU/cork test samples.

### 2.3. Energy Absorption Testing

**Figure 5:**
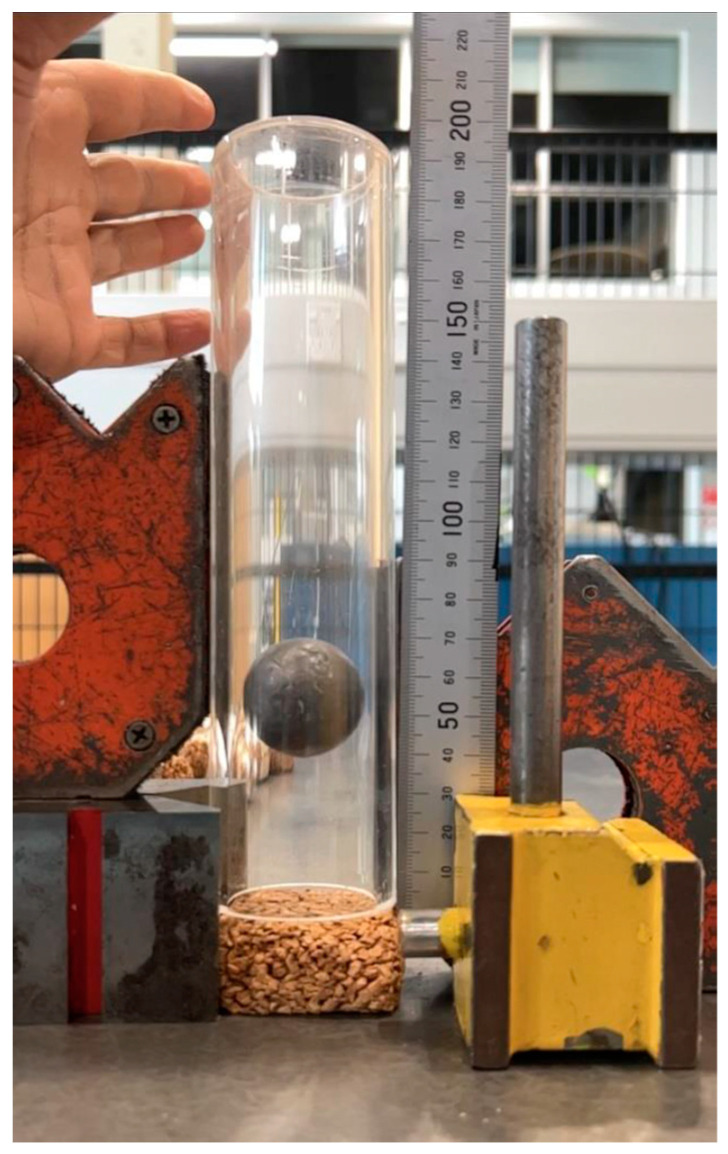
Energy absorption test rig and specimen showing steel ball at maximum rebound height.

A drop test was devised to measure the energy absorption of each of the test samples. This system entailed the release of a metal ball of mass 184.6 g from a height of 200 mm within an acrylic tube held above the test sample under consideration. The maximum rebound height of the ball, shown in Figure 5, was recorded by a high-speed camera and the energy absorbed during the impact was calculated by comparing the change in potential energy between the initial and maximum rebound heights.

### 2.4. Compression Testing

**Figure 6:**
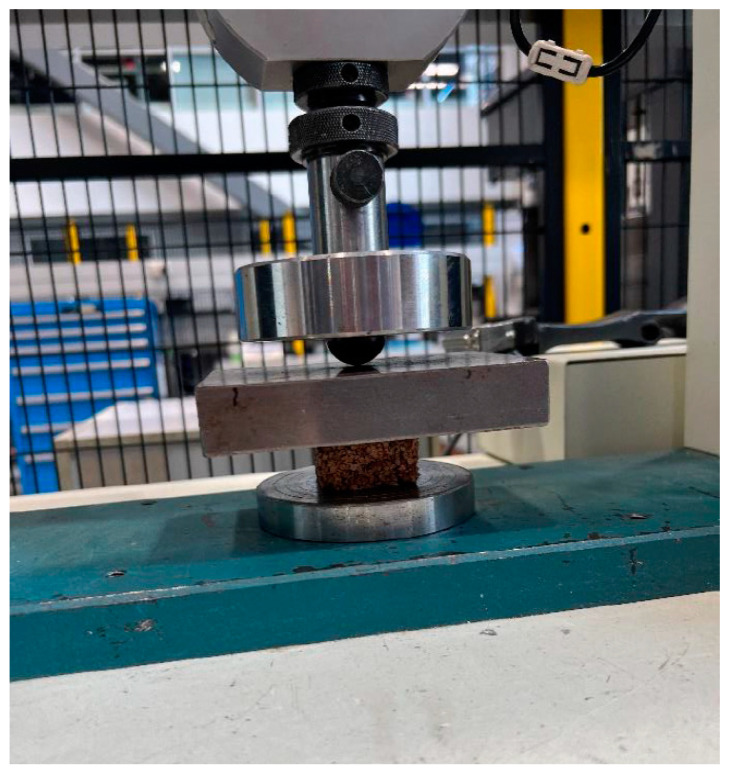
Test specimen placed between two flat thick steel plates within Tinius Olsen H50KS universal testing machine.

Each test sample was placed between two flat thick steel plates within a Tinius Olsen H50KS universal testing machine (Tinius Olsen Inc., Horsham, PA, USA), shown in Figure 6, where it experienced a progressively increasing compression force at a controlled rate of 3 mm/min.

Each test specimen experienced a progressively increasing compression force up to a maximum of 50 kN, which allowed us to generate force–displacement graphs from which the Young’s modulus of elasticity was calculated. The spring constant of each specimen was also determined from the slope of the force–displacement graph produced by the testing machine; however, it should be noted that this parameter is specific to the cross-sectional area under test.

## 3. Results

Initial mechanical analysis of the polyurethane/cork test samples demonstrated that changing setting pressures across the 5 kPa to 26 kPa range had no effect on any of the mechanical properties being tested; so, the results for each of the three samples set at different pressures were combined. This result agrees with the findings of another study where the binder setting pressure had no influence on the energy absorption characteristics [17]. This finding enabled our investigation to focus on the effects that changing the process variables of the binder/cork mix ratios and setting temperatures had on the desired mechanical properties.

### 3.1. Energy Absorption

**Figure 7:**
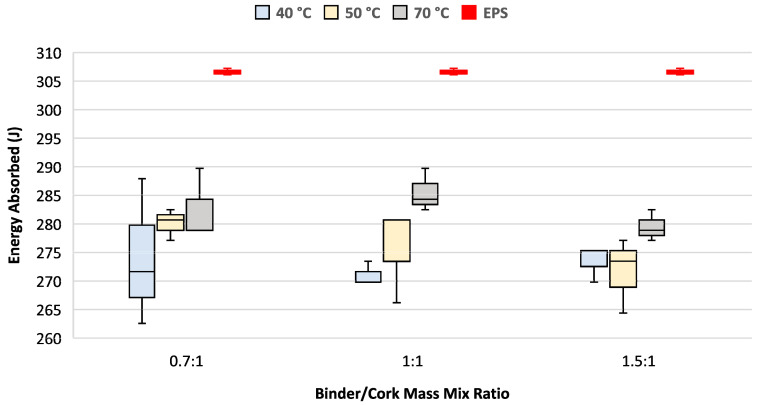
Energy absorption as function of curing temperature and binder/cork mix mass ratio. Helmet EPS sample test results shown in red.

A higher curing temperature was found to increase the amount of energy absorbed by each of the test samples independently of the binder/cork mix ratio, as shown in Figure 7. Increasing the binder/cork mix mass ratio did not change the mean energy absorption but produced more consistent results between the test samples.

All variations of the binder/cork mix mass ratio and curing temperature tested absorbed between 7.2% and 11.1% less energy than EPS.

### 3.2. Young’s Modulus of Elasticity

**Figure 8:**
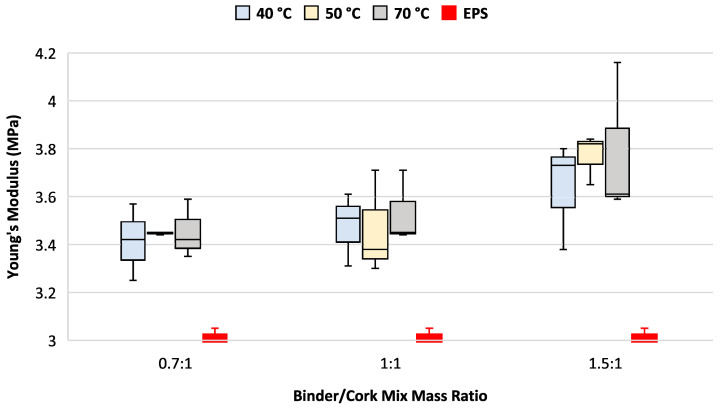
Young’s modulus as function of curing temperature and binder/cork mix mass ratio. Helmet EPS sample test results shown in red.

Changes in the setting temperature seemed to have little effect on Young’s modulus for each of the three binder/cork mix mass ratios under consideration; however, there is a trend of increasing stiffness when increasing the amounts of PU binder added, as shown in Figure 8. Interestingly, greater variation in Young’s modulus between the test samples was also found when increasing amounts of PU binder were used.

All variations of the binder/cork mix mass ratio and curing temperature tested demonstrated between a 9.8% and 24.6% higher Young’s modulus of elasticity compared to EPS.

### 3.3. Spring Constant

**Figure 9:**
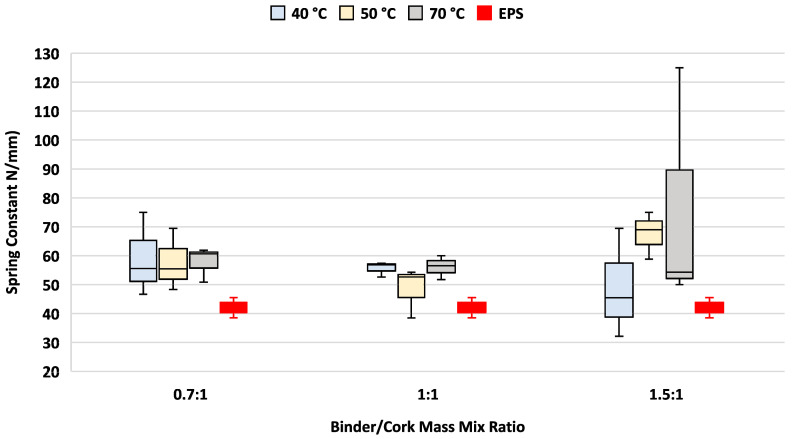
Spring constant as function of curing temperature and binder/cork mix mass ratio. Helmet EPS sample test results shown in red.

Variations in both the PU/cork mix mass ratio and setting temperature were both found to have little effect on the spring constant; however, there was greater variation in the spring constant results found between different curing temperatures when increasing amounts of binder were used, as shown in Figure 9.

All variations of the binder/cork mix mass ratio and curing temperature tested demonstrated between a 12% and 58% higher spring constant compared to EPS.

## 4. Discussion

The finding that changing the setting pressures across the 5 kPa to 26 kPa range had no effect on any of the mechanical properties being tested is consistent with another study where the binder setting pressure had no influence on energy absorption characteristics [17].

### 4.1. Energy Absorption

All PU/cork test samples demonstrated lower levels of energy absorption than those found for the EPS sample, with medians ranging from 7.2% to 11.1% lower. Analysis of the results shown in Figure 7 found that energy absorption levels increased with increasing curing temperature across all binder PU/cork mix mass ratios tested, suggesting that the PU binder’s mechanical characteristics were linked to curing temperature more strongly than the cork mechanical properties were. This finding agrees with another study that found resin’s nature and content to significantly influence the physical–mechanical properties when used as a binder in a cork matrix [15]. While the greatest energy absorption levels were achieved with the 1:1 PU/cork mix mass ratio that was cured at 70 °C, the energy absorption levels of the other two mix ratios were also very similar. Further testing with more samples is needed to demonstrate if there is any change occurring with different mix ratios.

### 4.2. Young’s Modulus of Elasticity

All PU/cork test samples demonstrated higher levels of stiffness, with medians ranging from 9.8% to 24.6% greater than those found for the EPS sample. Analysis of these results found that the elastic constant levels generally increased with increasing PU/cork mix mass ratios tested across all curing temperatures. The most elastic samples used the lowest amount of PU binder, having a 0.7:1 PU/cork mix mass ratio and curing temperatures ranging from 40 °C to 70 °C. While in our study, increasing curing temperature over the range tested produced no significant differences in the elastic modulus, another study has shown that increasing curing temperatures beyond 70 °C would lower Young’s modulus of elasticity [18] so that it becomes closer to that of EPS.

### 4.3. Spring Stiffness

It was not surprising that the spring stiffness results closely followed those of Young’s modulus of elasticity. The purpose of measuring this metric was to determine performance during compression testing. Like the elastic modulus, analysis of the spring stiffness found levels generally increased when increasing the PU/cork mix mass ratios tested across all curing temperatures tested. This result is not unexpected given the mechanical properties of PU tend to dominate when greater amounts of binder resin are used. Consistent with the elastic modulus findings, all PU/cork test samples demonstrated higher median levels of spring stiffness, ranging from 7.1% to 61.9% greater than those found for the EPS sample. The sample mixed at a 1.5:1 binder/cork ratio and cured at 40 °C displayed the closest spring stiffness to that of EPS. Analysis of data trends suggests that spring stiffness levels remain relatively constant across all curing temperatures and binder PU/cork mix mass ratios tested. Greater variation in spring stiffness results across the PU/cork test samples was found at the highest (1.5:1) mix mass ratio.

### 4.4. Process Variables

No change in any of the mechanical parameters being tested in this investigation were found over the range of setting pressures tested. Our study suggests that exerting pressure on the setting binder/cork matrix only serves to exclude air from the sample pieces and enables complete filling of the mould, rather than changing any mechanical properties. This finding enabled the results from this pilot study for the different setting pressures to be merged, enabling this study to have three samples to test each of the other two process variables: the binder/cork mix ratio and setting temperature.

Over the range of variables tested, increasing the binder/cork mix ratio did not change the amount of energy absorbed by the matrix structure, which confirms earlier studies that also found that the amount of binder has no influence on energy absorption characteristics [17]. This finding suggests that the cork mechanical properties dominate within the matrix; however, the 7.2% to 11.1% lower energy absorption characteristic would need to be taken into consideration during the design of any micro-transport helmet using this liner material. Agglomerated cork is characterised with a Young’s modulus of elasticity much lower than that of natural cork [19], and with polyurethane resin having a much higher value of Young’s modulus of elasticity [20], so it is not surprising that increasing the amount of binder within the matrix leads to a stiffer structure, as shown in Figure 8.

Out of the three process variables tested, curing temperature and the binder/cork mix mass ratio had the greatest influence, but for different reasons. Here, higher temperatures cause increasing energy absorption but lead to a stiffer structure, potentially caused by temperature-induced change in binder cross-linking during the curing process, which requires further investigation. Temperature-elicited change in the liquid binder rheological properties [21] may also have played a role here by changing the penetration of PU into the cork during the curing process. Increasing the binder/cork mix mass ratios led to higher Young’s modulus values due to PU having a higher elastic modulus than that of cork; however, further testing is required to find the limit of binder reduction before the matrix structural integrity becomes compromised.

There is also the effect that changing the agglomerated cork particle size would have on the energy absorption and elastic properties of the PU/cork matrix. While this process variable was not explored in this investigation, other studies have found that a reduction in cork particle size leads to more compliant and energy-absorbing composite matric materials [13]. This assumption needs to be validated in a future study.

### 4.5. Suitability for Micro-Transport Helmets

While the mechanical properties of energy absorption, Young’s modulus of elasticity, and spring stiffness found in this study do not match those of EPS, the differences between these values and the current benchmark suggest that the polyurethane/agglomerated cork matrix could be used as an energy-absorbing and structural component in a suitably designed micro-transport helmet if changes were made to accommodate this stiffer material. These could include using this PU/cork matrix material as a lattice helmet liner as it has been shown that this configuration has an excellent capability of reducing peak rotational acceleration and outperforms the protection levels offered by existing EPS for single impacts when made from PA12 (nylon-12) [22], a 3D-printed material characterised by less energy-absorbing capabilities and a higher elastic modulus than the PU/cork matrix used in this study.

### 4.6. Limitations

In-depth statistical analysis of the results was not undertaken in this pilot study due to the small number of samples tested for each process variable (*n* = 3). The purpose of this investigation was to identify process variables that influence the mechanical properties of the urethan/agglomerated cork matrix which then could inform the design of another suitably powered study.

## 5. Conclusions

Curing temperature and the binder/cork mix mass ratio both influenced the mechanical properties of energy absorption, Young’s modulus of elasticity, and spring stiffness of a polyurethane/agglomerated cork matrix.

While the desired mechanical properties do not match those of EPS, the lower performance of the PU/agglomerated cork matrix could be accommodated through re-=design of current helmet structures to provide equivalent protection to that found in current micro-transport helmets.

Reducing the size of cork particles used in this study may result in mechanical characteristics more comparable to those of EPS, which could then enable the PU/cork matrix material to be used as a more ecological direct replacement for EPS.

The pilot results from this study justify further investigation, using a larger sample size and a small cork particle size, into the effects that process variables for a PU/agglomerated cork matrix have on its mechanical properties in relation to the desired applications of micro-transport helmets.

## Figures and Tables

**Table 1:** List of process variables used to create test samples.

Binder Mass (g)	Cork Mass (g)	Mix Ratio	Setting Force (kg)	Setting Pressure (kPa)	Curing Temperature (°C)
3.5	5.0	0.7:1	1	5.3	40
3.5	5.0	0.7:1	2	10.6	40
3.5	5.0	0.7:1	5	26.5	40
3.5	5.0	0.7:1	1	5.3	50
3.5	5.0	0.7:1	2	10.6	50
3.5	5.0	0.7:1	5	26.5	50
3.5	5.0	0.7:1	1	5.3	70
3.5	5.0	0.7:1	2	10.6	70
3.5	5.0	0.7:1	5	26.5	70
5.0	5.0	1:1	1	5.3	40
5.0	5.0	1:1	2	10.6	40
5.0	5.0	1:1	5	26.5	40
5.0	5.0	1:1	1	5.3	50
5.0	5.0	1:1	2	10.6	50
5.0	5.0	1:1	5	26.5	50
5.0	5.0	1:1	1	5.3	70
5.0	5.0	1:1	2	10.6	70
5.0	5.0	1:1	5	26.5	70
7.5	5.0	1.5:1	1	5.3	40
7.5	5.0	1.5:1	2	10.6	40
7.5	5.0	1.5:1	5	26.5	40
7.5	5.0	1.5:1	1	5.3	50
7.5	5.0	1.5:1	2	10.6	50
7.5	5.0	1.5:1	5	26.5	50
7.5	5.0	1.5:1	1	5.3	70
7.5	5.0	1.5:1	2	10.6	70
7.5	5.0	1.5:1	5	26.5	70

## Data Availability

Data are contained within the article.
